# Exposure time reduction of secondary radiographs used in digital subtraction radiography in detecting intrabony change

**DOI:** 10.1007/s11282-013-0134-4

**Published:** 2013-04-04

**Authors:** Yukiko Matsuda, Tsuneyasu Terauchi, Kota Murahira, Seema Patil, Vajendra Joshi, Kazuyuki Araki, Akira Taguchi, Tomohiro Okano

**Affiliations:** 1 Division of Radiology, Department of Oral Diagnostic Sciences, Showa University School of Dentistry, 2-1-1 Kita-senzoku, Ohta-ku, Tokyo, 145-8515 Japan; 2 Department of Biomedical Engineering, Tokyo City University, 1-28-1 Tamazutumi, Setagaya-ku, Tokyo, 158-8557 Japan

**Keywords:** Intraoral radiography, Digital subtraction radiography (DSR), Exposure time reduction, Charge-coupled device (CCD)

## Abstract

**Objectives:**

Digital subtraction radiography (DSR) is a suitable technique for detecting incipient bone changes. However, in DSR, one or more follow-up radiographs must be taken. The aim of this study was to assess the possibility of reducing the exposure time for the radiographs that follow the initial one.

**Methods:**

Maxillary premolar and molar radiographic images of a dry skull were taken with a digital radiography system. The initial radiographs, without bone chips, were taken at 0.32 and 0.16 s. Then, five bone chips (weight range 7–15 mg) were placed on the maxillary molar buccal side of the dry skull. Secondary radiographs were taken at 0.32-, 0.16-, 0.08-, 0.04-, and 0.02-s exposure times. For each bone chip, radiographs were taken three times. The secondary and initial images were subtracted to yield subtraction images. Four observers were asked to evaluate bone change visibility in the subtraction images. The Friedman test was used for statistical analysis.

**Results:**

Significant differences were seen at each of the settings for the 0.32-s group (*p* = 1.24e−030) and 0.16-s group (*p* = 7.52e−009). By comparing the different groups, observer evaluations indicated that visibility changed when the secondary radiograph was taken at 1/8 of the exposure time of the initial radiograph. In both groups, the visibility of the 0.02-s subtraction image was significantly lower than that of the other subtraction images.

**Conclusion:**

In DSR, the exposure time of the secondary radiograph can be reduced to 1/4 of the exposure time of the initial radiograph.

## Introduction

Radiography is the main diagnostic tool for the assessment of hard tissue changes. Periapical radiographs clearly show bone trabeculation, the periodontal ligament space, and the lamina dura. However, some anatomical structures are difficult to distinguish on two-dimensional radiographs, so it may be difficult to assess the presence of incipient bone changes.

Digital subtraction radiography (DSR), in which identical features in a series of radiographs taken at different times are eliminated, makes it easier for the physician to spot lesions and changes. This is because the only features that remain on the final image (known as the “subtraction image”) are areas that have changed between the initial and the follow-up (secondary) images. Several subtraction programs have been created [1–3] and assessed [4–8]. Reports about these programs have noted the effectiveness of this technique for visualizing alveolar bone changes. The utility of DSR for assessing furcation defects [6], peri-implantitis [7], and immediate loading implant treatment [8] has also been assessed.

Digital dental radiographic systems have several benefits: no developing process is necessary; image processing is easier than with normal films; and a reduced exposure time is possible [9]. In previous studies, it was confirmed that the exposure time can be reduced to 1/8 of that of D-speed film in an in vivo study on interproximal caries diagnosed using an imaging plate system and a charge-coupled device (CCD) system [10–12].

Recently, Stephanopoulos et al. [13] assessed simulated internal resorption cavities using DSR with a CCD and concluded that DSR is superior to normal digital radiography for the detection and progress monitoring of internal root resorption.

For the application of DSR, several secondary radiographs have to be taken to determine its ability to visualize small bone changes. DSR with film has been well analyzed. Because digital radiography is widely replacing film radiography, we are interested in analyzing its application to subtraction radiology (i.e., DSR). One of the advantages of a digital system is the ability to reduce exposure time as compared with film. If the purpose of taking a follow-up radiograph is just to detect hard tissue changes, then we would like to know how much to reduce the radiation exposure time by using digital radiography, and how much exposure time reduction will be available for a secondary radiograph. To our knowledge, there has been no research on DSR with regard to exposure reduction for the secondary radiograph.

In this study, we aimed to determine if DSR using digital dental radiographs instead of film would allow the physician to reduce the exposure time of radiographs taken after the initial radiograph.

## Materials and methods

**Fig. 1:**
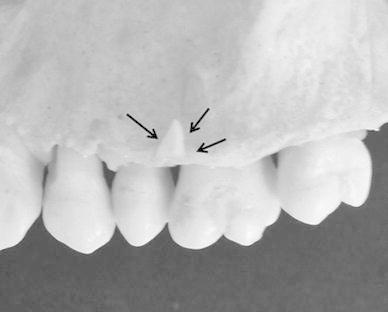
An example of the dry skull with a placed bone chip. The bone chip (*arrows*) was placed on the facial alveolar bone surface of the interproximal area between the second premolar and first molar to simulate bone gain

One dry skull from a young adult cadaver with a complete set of teeth was used in this study. The skull was obtained more than 15 years ago, and it is impossible to identify the donor. Five small bone chips (weight range 7–15 mg, thickness 1 mm), which were obtained from cortical bone of the cervical spines of dairy cows, were used to simulate bone changes. The weight of each bone chip is given in Table 1. These bone chips were confirmed to detect bony changes by subtraction analysis with film. Each bone chip was placed on the buccal surface of alveolar bone around the alveolar crest of the maxillary premolar or molar teeth of the dry skull. Figure 1 shows an example of the dry skull with a placed bone chip. In this sample image, the bone chip was placed on the facial alveolar bone surface of the interproximal area between the second premolar and the first molar.

**Table 1:** Weight of the bone chips

Bone chip	Weight (mg)
A	15
B	10
C	7
D	11
E	10

An RVG 5000 CCD system (Kodak, Rochester, NY, USA) was used in this study. We used the size 1 sensor of the system, which has external dimensions of 40 × 27 mm, a pixel matrix of 1200 × 1600, and a resolution of 14 lines/mm. This sensor is capable of capturing 4096 shades of gray. Images were acquired using the bundled Kodak Dental Imaging Software.

**Fig. 2:**
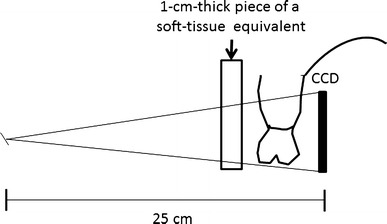
Exposure geometry was maintained constant. The distance between the focus and the sensor was 25 cm. A 1-cm-thick piece of a soft-tissue equivalent, placed between the object and the source, served as a simulation of human tissue. These settings simulate the clinical conditions

Exposure was set at 60 kV and 7 mA (HD-70 X-ray generator; Asahi Roentgen, Kyoto, Japan). The CCD sensor was set behind the upper molar and premolar teeth. The focus-to-sensor distance was set at 25 cm. The presence of soft tissue was simulated by placing a 1-cm-thick piece of a soft-tissue equivalent material (Tough Water Phantom; Kyoto Kagaku, Kyoto, Japan) in the appropriate place. Figure 2 shows the exposure geometry.

Initial radiographs were taken at 0.32 and 0.16 s without any bone chips in place. These exposure times were selected because they are equal to and half that of the normal exposure time required for E/F-speed film, respectively. Secondary radiographs were taken with bone chips in place and exposure times of 0.32, 0.16, 0.08, 0.04, and 0.02 s. For each bone chip, radiographs were taken three times. This resulted in a total of two images taken without bone chips, which were the initial images, and 75 images with bone chips, which were the secondary images. These images were exported as Digital Imaging and Communication in Medicine (DICOM) files and converted to 8-bit TIFF files for the subtraction analysis.

In this study, we used the subtraction radiography program previously reported by Murahira and Taguchi [14]. This software uses a novel method to accurately extract suitable corresponding anatomical points from two images to achieve an exactly matching pair. The software automatically selects points of interest in the initial image and these points are then detected in the secondary images. The methods to extract suitable feature points from the reference image based on the premise of the corresponding points are obtained from the objective image accuracy. The secondary images are then superimposed on the initial image. Next, a normalization step is performed that eliminates brightness and contrast differences between the two images. Finally, the two images are superimposed and subtracted automatically. The subtracted images are saved in TIFF format.

**Fig. 3:**
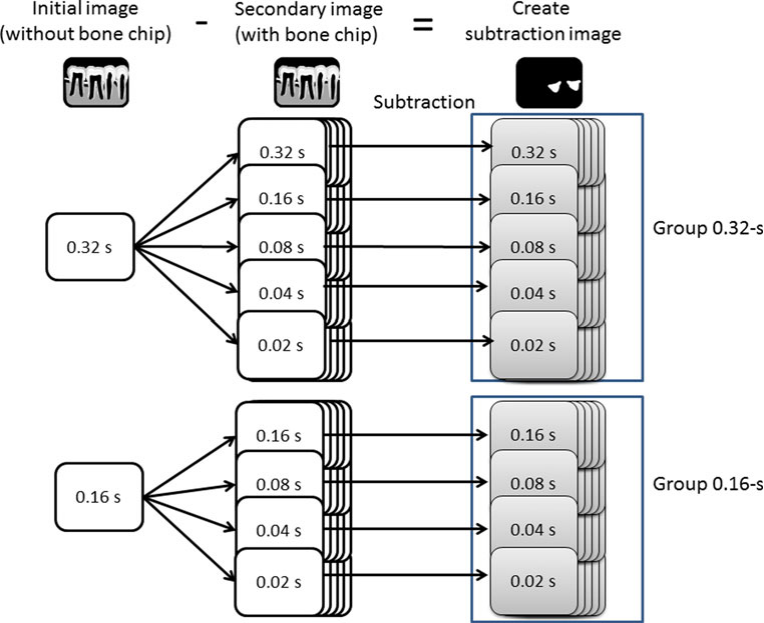
Created subtraction image group consisting of the 0.32-s group and the 0.16-s group with their subgroups. The 0.32-s group was created by subtracting the secondary radiographs that were taken with exposure times of 0.32, 0.16, 0.08, 0.04, and 0.02 s from the initial radiograph that was taken at 0.32 s. The 0.16-s group was created by subtracting the secondary images that were taken with exposure times of 0.16, 0.08, 0.04, and 0.02 s from the initial radiograph that was taken at 0.16 s. The 0.32-s group consisted of five subgroups, and the 0.16-s group consisted of four subgroups

In this study, two kinds of subtraction image groups were created: (1) the 0.32-s group, which was created by subtracting the secondary radiographs that were taken with exposure times of 0.32, 0.16, 0.08, 0.04, and 0.02 s from the initial radiograph that was taken at 0.32 s; and (2) the 0.16-s group, which was created by subtracting the secondary images that were taken with exposure times of 0.16, 0.08, 0.04, and 0.02 s from the initial radiograph that was taken at 0.16 s. The schema for the subtraction image group, which consists of the 0.32-s group, the 0.16-s group, and their subgroups, is given in Fig. 3.

The resulting subtraction images are referred to in this paper as follows: a subtraction image created by subtracting a secondary radiograph with an exposure of 0.16 s from an initial radiograph with an exposure of 0.32 s is referred to as a 0.32/0.16 subtraction image (i.e., the 0.16-s radiograph is subtracted from the 0.32-s radiograph). In this fashion, the following subgroups were created: (1) 0.32/0.32, 0.32/0.16, 0.32/0.08, 0.32/0.04, and 0.32/0.02; and (2) 0.16/0.16, 0.16/0.08, 0.16/0.04, and 0.16/0.02.

The 0.32-s group consisted of five subgroups, and the 0.16-s group consisted of four subgroups. Each subgroup consisted of 15 subtracted images. A total of 75 subtracted radiographic images were obtained for the 0.32-s group and a total of 60 subtracted radiographic images for the 0.16-s group.

Four experienced dentists (one general dentist and three oral radiologists, each with experience of 20–30 years) assessed the images. They were also required to have good knowledge of the effects of image manipulations. During the viewing sessions, the observers were asked to evaluate the visibility of bone changes on the following five-point scale: 1, visible; unnecessary anatomical structures on a radiographic image were eliminated and clearly highlighted the bone change area; 2, possibly visible; unnecessary anatomical structures were eliminated on a radiographic image but the margin of the bone change area was slightly unclear; 3, fair; unnecessary anatomical structures were eliminated on a radiographic image but the margin of the bone change area was unclear; 4, poor; unnecessary anatomical structures were not eliminated on a radiographic image but the bone change area was visible; 5, not acceptable; it was impossible to visualize the bone changes. Thus, 60 pieces of assessment data (15 subtraction images × 4 observers) were obtained for each subgroup. The subtraction images that were obtained from the secondary radiograph with the same exposure time as the initial radiograph were used as the gold standard.

To find the limit of exposure time reduction on secondary radiographs, the Friedman test was performed using statistical analysis software (SPSS, version 14; SPSS Inc., Chicago, IL, USA). The significance level was set at 0.05.

## Results

**Fig. 4:**
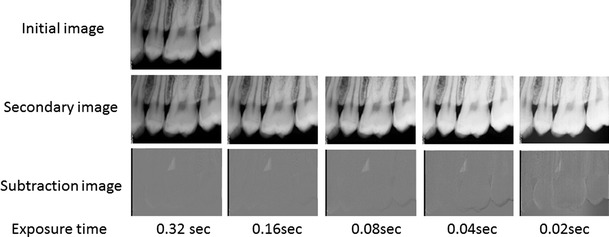
Examples of initial, secondary, and subtraction images in the 0.32-s group. The secondary images were taken with a 10-mg bone chip. *Top row* initial radiographic image. *Middle row* secondary radiographic images taken at various exposure times. *Bottom* *row* subtraction images created by subtracting the secondary images from the initial image. The anatomical structures remained visible on the subtraction images prepared from radiographs taken with exposure of 0.04 s, but the areas of bony change were no longer visible with exposure of 0.02 s

Examples of an initial radiograph, secondary radiographs for each exposure setting, and subtracted radiographs are shown in Fig. 4 (0.32-s group). The anatomical structures remained visible, and the areas of bony change were sometimes invisible in the subtraction images created with secondary radiographic images that were taken at exposures of 0.04 and 0.02 s.

The results for the visibility of bone changes in each group are given in Table 2. In this study, a lower number indicates better visibility of the bone changes in the subtraction image. In both groups, the visibility of the 0.02-s subtraction image was different from that of the subtraction images obtained from the secondary radiograph with the same exposure time as the initial radiograph.

**Table 2:** Frequency of the visibility of bone changes on the five-point scale

Group/Exposure time (s) of secondary radiograph	Five-point scale				
	1	2	3	4	5
0.32-s group					
0.32	57	3	0	0	0
0.16	54	5	1	0	0
0.08	53	6	1	0	0
0.04	34	7	12	1	6
0.02	6	9	8	6	31
0.16-s group					
0.16	57	3	0	0	0
0.08	55	5	0	0	0
0.04	46	8	6	0	0
0.02	7	9	10	3	31

By comparing the different subgroups, it can be seen that the visibility changed when the secondary image was taken with an exposure of less than 0.04 s. The visibility was clearly lower than that of the other subgroups when the exposure time of the secondary radiograph was reduced to 0.02 s

*1* visible, *2* possibly visible, *3* fair, *4* poor, *5* not acceptable

**Fig. 5:**
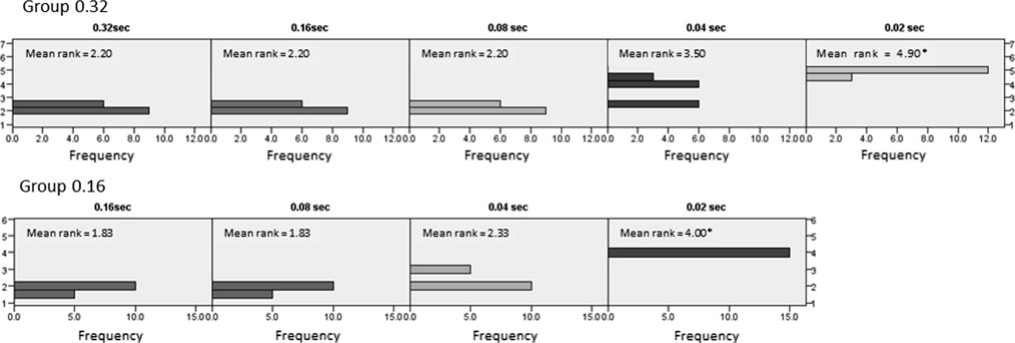
The mean rank and the frequency of the ranking of the visibility. A lower mean rank indicates better visibility. Using the Friedman test, the mean rank of the 0.02-s secondary subtraction images was lower than that of the other secondary subtraction images

Figure 5 shows the mean rank and the frequency of the visibility of each setting. The Friedman test was used to test for differences between groups when the dependent variable being measured was ordinal. A lower number rank means better visibility. Significant differences were seen in each of the settings for the 0.32-s group (χ^2^ = 146.33, *p* = 1.24e−030) and 0.16-s group (χ^2^ = 40.71, *p* = 7.52e−009). In comparisons with the rank of 0.02 s, it was clearly lower than any other exposure time.

## Discussion

To our knowledge, this is the first report on the possibility of reduced exposure times for secondary radiographs during DSR. This opens the possibility of a new era in the use of intraoral digital systems. For DSR analysis with film, the conditions under which the processing is done and the developed films are digitized must be carefully monitored. By using digital systems, we can eliminate these processes.

In this study, the exposure time of secondary radiographs can be safely reduced by up to 1/4 of the exposure time of the initial radiograph, which was taken at the same exposure time required for E/F-speed film, maintaining the possibility of detecting bone changes by DSR. Significant differences were seen in both groups using the Friedman test. The SPSS program that we used for analyses does not have a post hoc test program. This lack is a limitation of the study, as it was impossible to evaluate which of the subgroup scores were different from the others. However, the visibility and the mean rank were clearly lower than those of other subgroups when the exposure time of the secondary radiograph was reduced to 0.02 s.

Regarding the possibility of reduced exposure in a digital system, we previously assessed a CCD sensor (RVG-UI CCD system; Kodak) [10]. In that study, for normal radiographs, we used Kodak InSight film and exposure was adjusted to that of E-speed film, whereas for the RVG, we reduced the exposure speed to half that of E-speed film. We concluded that there were no significant differences between the two systems. We could not directly compare the results obtained using the RVG-UI sensor with those from the RVG 5000 system, but they suggest that the sensor becomes more sensitive as the exposure time is reduced.

In this study, the 0.32-s group subtraction images created with an exposure time of 1/8 of the initial radiograph (0.04 s) showed anatomical structures but the area of the bone chip was clearly discernible. However, when the exposure time was reduced to 1/16 of the initial exposure time (i.e., 0.02 s), sometimes bone changes were no longer visible in the subtraction image. In the 0.16-s group, subtraction images that were created with secondary images reduced to 1/8 of the exposure time of the initial image (i.e., 0.02 s) did not show bone changes. The limited visibility of bone changes on subtraction images taken at these reduced exposures may be related to the exposure time of the secondary radiographs. An exposure time of 0.02 s is too short for the secondary radiographs. It may be possible to further reduce the exposure time for the initial image, but if it is reduced by too much, the image becomes “noisy”. Even with short exposure times, digital radiographs contain noise of various levels, which can arise from fluctuations in X-ray photons, low radiation doses, or instability/deficiencies in the electronics of the detectors. On the reduced exposure time images, the image-to-noise ratio could increase and this may limit how far exposure times can be reduced while still yielding useful images.

In the 0.32-s group, the mean ranks of the visibility of the 0.32/0.32, 0.32/0.16, and 0.32/0.08 subtraction images were the same. However, the mean ranks of the visibility of the 0.32/0.04 and 0.32/0.02 subtraction images differed from that of the 0.32/0.32 subtraction image. In the 0.16-s group, the mean ranks were the same for the 0.16/0.16 and 0.16/0.08 subtraction images. However, when the exposure time of the secondary radiographs was less than 0.08 s, the mean ranks of the subtraction images changed. Scores 4 and 5 were only given for exposure times of less than 0.08 s.

In this study, we used the DSR software created by Murahira and Taguchi [14] because it yields good reproducibility of the subtraction image. When using this system, two radiographs are registered automatically and the resulting subtraction image is free from observer bias. This software utilizes the histogram-matching contrast correction method introduced by Gonzalez and Woods [15]. This method is capable of producing corrected images with relatively small contrast deviations from the reference image in each set. With this program, the background image is completely subtracted and only the bone chip remains visible. Murahira and Taguchi compared their method with other subtraction methods and found that their method was superior to other methods that extract feature points from the reference image and corresponding points on the secondary image.

Contrast correction is necessary in DSR, and several contrast correction methods have been introduced and applied. Versteeg and van der Stelt [16] assessed the logarithmic contrast enhancement method. Economopoulos et al. [17] used the histogram registration method, whereas Hildebolt et al. [18] used the histogram-matching and histogram-flattening contrast correction method. These methods may be better to subtract for incipient bone changes, but in this study the quality of the subtracted images was sufficient to visualize bone changes. With an improved method of contrast correction, further reductions in exposure times for DSR may be possible without losing visibility of bone changes.

In this study, 7- to 15-mg bone chips were used. Previously, quantification of changes in terms of milligram equivalents by DSR has required the use of bone chips or other intraoral standards. Couture and Hildebolt [19] assessed the ability of an imaging plate system to detect bone changes. They concluded that the detection limit was 0.02 g/cm^2^ for large image areas (more than 7 mm^2^) and 0.3 mg for areas of 1 mm^2^ or smaller. Bragger [20] used DSR to measure the mass of bone chips up to 6 mg. Byrd et al. [21] used bone chips weighing less than 10 mg. They concluded that when used with a subtraction program with four-point affine warp algorithms, the sensitivity and specificity were 100 %. It was not clear from their report what the minimum weight was for the bone chips that they studied.

In the near future, we will assess the limits of bone chip size that can be detected with DSR using a digital system. Furthermore, we will compare various DSR programs using the same digital radiographs and find the lower limit to which exposure times can be reduced for each of these programs.

In conclusion, in DSR, the exposure time of the secondary radiograph can be reduced to 1/4 of the exposure time of the initial radiograph while still maintaining sufficient image quality. It is difficult to find a simple explanation for the results because of the many factors involved (e.g., digital system and observers’ ratings).
